# Knee kinematics and kicking distance: an IMU and OpenSim-based cross-sectional study

**DOI:** 10.3389/fspor.2025.1605545

**Published:** 2025-07-10

**Authors:** Wangyang Xu, Bo Gong, Xinbi Zhang, Diyan Zhang

**Affiliations:** ^1^ School of Athletic Performance, Shanghai University of Sport, Shanghai, China; ^2^ School of Kinesiology and Health, Capital University of Physical Education and Sports, Beijing, China; ^3^ Physical Education Institute, Henan Normal University, Xinxiang, China

**Keywords:** football, kicking, knee kinematics, inertial measurement units, OpenSim

## Abstract

This study aims to examine the correlation between knee joint kinematics and kicking distance in soccer players across different kicking phases. Twenty-six soccer players participated in the testing for this study. The lower limb posture data for each participant were collected using IMUs, and modeling analysis was conducted using OpenSim. During the approach phase, the extremum angle of the second knee flexion (*r* = 0.152, *p* = 0.041), as well as the ROM of the second knee extension (*r* = 0.169, *p* = 0.023) and the average angular velocity of the second knee extension (*r* = 0.185, *p* = 0.013), were positively correlated with the kicking distance. During the swing phase, the extremum angle (*r* = 0.178, *p* = 0.016) and the average angular velocity (*r* = 0.283, *p* < 0.001) of knee extension were positively correlated with the kicking distance. The findings suggest an association between specific knee kinematic patterns and the ability to achieve longer kicking distances. These kinematic patterns are characterized by: larger flexion angle during the ground contact phase of the approach; faster extension velocity and greater extension during the push-off; as well as rapid extension velocity and a larger final flexion angle during the swing.

## 1 Introduction

Current research predominantly focuses on exploring soccer ball velocity, particularly its relationship with lower limb biomechanical characteristics (1–3). The key to increasing ball velocity lies in optimizing limb movement patterns and enhancing the efficiency of velocity transfer from the foot to the ball (4). The kicking motion exhibits a typical proximal-distal timing characteristic: the thigh (the proximal segment) initiates the forward swing first, driving the delayed explosive movement of the lower leg and foot (the distal segments) (4). The knee joint, serving as the core hub of this kinetic chain, plays a crucial role in power transmission and velocity generation (5). Naito et al. developed a 3-D dynamical model of the multi-joint kinetic chain in instep kicking to quantify the contributions of key dynamical factors to maximizing knee extension angular velocity (6). Their findings highlighted that the leg structure (knee angle) is important for effective instep kicking. Moreover, Sinclair et al. reported that sagittal plane knee extension angular velocity is significantly related to foot linear velocity for instep kicking (7). However, existing research mainly focuses on the biomechanics of the kicking leg during the swing before and after ball contact (8). The kicking action is a complex, multi-phase movement that requires the coordination of both lower limbs. The complete kicking process includes three key phases: the approach run, the positioning of the supporting foot, and the final phase of leg swing and ball contact. The effectiveness of the kicking action is dependent on the coordination of each phase (9). Therefore, although knee angle and velocity have been proven to be important parameters affecting kicking performance (5), their specific roles at various phases of the movement have not been fully studied. Understanding these roles is crucial not only for maximizing ball velocity, but also for other performance metrics.

Indeed, while ball velocity is critical, the ability to execute long-range kicks represents another dimension of kicking performance with significant tactical value. Players can break through the opponent's offside trap and disrupt the defensive formation with long-range passes, creating defensive gaps. However, few biomechanical studies have focused on the distance of the kick. The majority of current studies have been conducted in laboratory settings, where the kicking target is placed only 1.6–4 m from the kicking point (8, 10, 11). In terms of research techniques, current studies usually conduct kinematic analysis using optical or image-based motion capture technologies, both of which have issues such as light interference, object occlusion, and limited applicability in field settings (12). These limitations have posed challenges for the comprehensive analysis of soccer technique research. With the innovative development of inertial navigation technology and micro-electromechanical systems, Inertial Measurement Units (IMUs) offer more possibilities for field research in soccer. OpenSim modeling analysis based on IMU data has been shown to yield knee angles that are consistent with those from optical motion capture, with a root mean square difference of 3–6 degrees (13).

Therefore, this study aims to analyze the correlation between kinematic parameters of the knee joint and kicking distances in soccer players across different phases of the kicking motion, by utilizing IMUs and OpenSim modeling.

## 2 Materials and methods

### 2.1 Participants

This study estimated sample size with G*Power 3.1.9.7, selecting the “Correlation: Bivariate normal model” option, with 0.3 for the coefficient of determination, 0.05 for the *α*-level, and 0.8 for the power (14). The calculation results indicated that the minimum sample size needed was 23 participants. Based on this, the study recruited a total of 26 elite level soccer players (15), comprising 13 males and 13 females, with an average age of 19.62 ± 0.85 years, an average height of 171.19 ± 8.03 cm, an average weight of 63.77 ± 9.48 kg, and an average training experience of 8.46 ± 2.16 years. All participants had the right leg as their dominant kicking leg and no history of lower limb injuries in the past year.

### 2.2 Procedures

**Figure 1:**
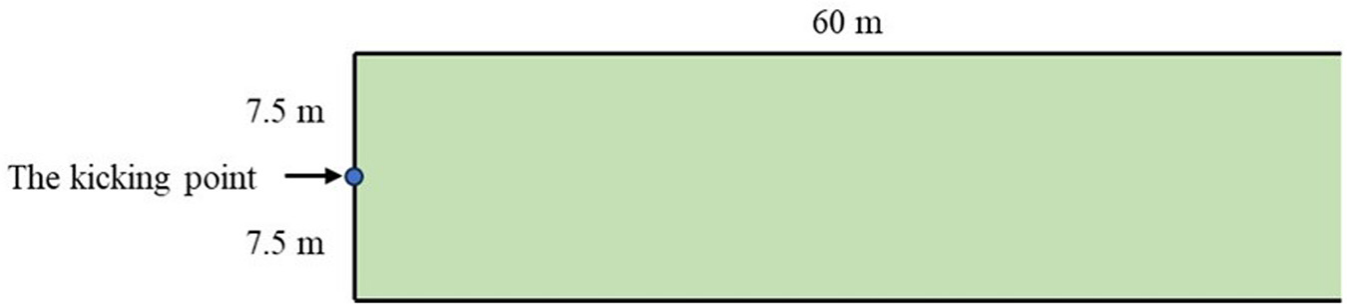
Schematic diagram of experimental site layout.

The experimental setup is shown in Figure 1. A 15 m line was drawn at an appropriate position on the soccer field to define the transverse width of the kicking area. From both ends of this line, two parallel lines, each over 60 m long, were drawn perpendicular to the field to define the longitudinal length of the kicking area, with distance markers indicated. Participants were instructed to stand behind the test line, place the soccer ball stationary at the midpoint, which was the designated kicking point, and freely choose a suitable approach route. They were to take one step with their right leg, land with their left leg to support their body, and use the inner side of their right foot's instep as the contact point to kick the ball with maximum force towards the farthest possible distance.

Researchers recorded the distance of each kick. The kicking distance is defined as the length from the kicking point to the landing point of the ball. One researcher is positioned at the kicking point to anchor one end of the measuring tape, while two others extend the tape to measure and record the distance. The smallest unit of measurement on the tape is 1 cm. If the landing point of a kick was outside the transverse boundaries of the kicking area, the result was considered invalid. The order of participation in the experiment was predetermined through randomization. To avoid fatigue, participants rested for 45 s between each kick until 7 valid kicking distance measurements were collected. This study collected a total of 182 valid kicking distances for analysis.

**Figure 2:**
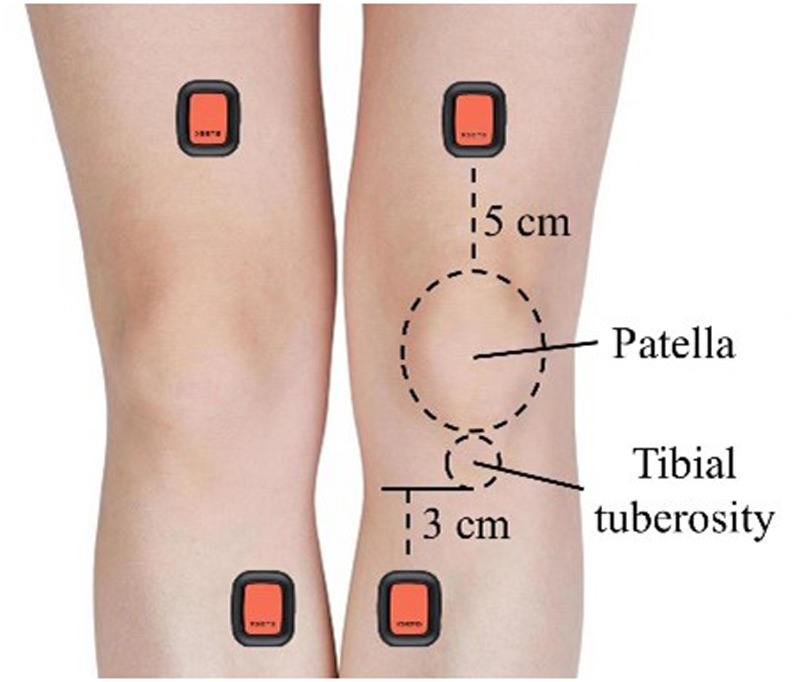
Schematic diagram of IMUs placement positions.

This study used four IMUs (Xsens Dot, Xsens Technologies, Netherlands) worn on the participants’ left and right thighs and shins to collect kinematic data of the corresponding limbs at a sampling frequency of 120 Hz. The specific wearing positions are illustrated in Figure 2. For instance, on one leg, one IMU was placed approximately 5 cm above the patella on the midline of the thigh, and the other was positioned on the medial side of the tibia, about 3 cm below the tibial tuberosity. IMUs were placed on areas with minimal fat and muscle, and secured with skin membranes and muscle tapes. This setup minimized errors caused by relative motion between the IMUs and the body's soft tissues, and effectively avoided unnecessary interference with the participants’ athletic performance. Previous research has confirmed that the IMU placement does not affect the reliability and validity of the data results (16). Before each test, researchers activated the IMUs for data collection and instructed the participants to maintain a vertical, stationary posture with their feet shoulder-width apart and their knees fully extended to calibrate the initial position of the knee joint.

### 2.3 Data processing

This study utilized OpenSim 4.4 (Stanford, USA) to convert the IMU data into a motion model. The “gait2392simbody.osim” model was loaded, and the “IMU Placer Tool” was used to import the IMU data from the static calibration, with the Euler angle rotation sequence set to (0, 0, 90). The model calibration was then completed. The “IMU Inverse Kinematics Tool” was selected, with the same Euler angle rotation sequence as above, and the IMU data from the kicking process was imported. The output data were saved in the “sto” format. The knee joint angle was defined as 0° when the knee was fully extended, where increasing joint angles indicate knee flexion and decreasing joint angles indicate knee extension.

**Figure 3:**
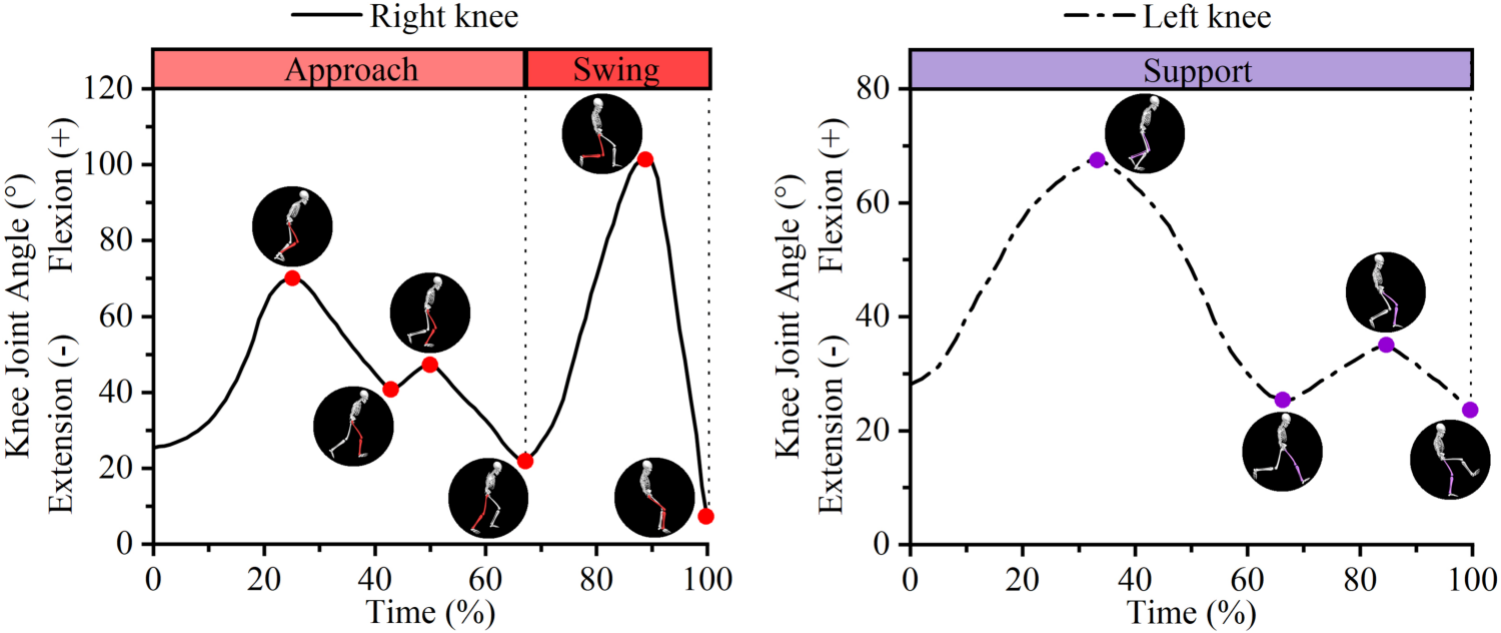
Schematic diagram of movement phase division in kicking technique.

As shown in Figure 3, this study classified the kicking technique into three phases: approach, support, and swing. Both the approach and support phases involved two knee flexion-extension movements (1st KF, 1st KE, 2nd KF, and 2nd KE), while the swing phase involved one (KF and KE). The extremum angle, the range of motion (ROM), and the average angular velocity during each knee flexion and extension were analyzed to evaluate the kinematic characteristics of the knee during the players’ kicking motion. The extremum angle refers to the maximum or minimum values that the knee joint angle reaches during flexion or extension. The ROM refers to the range of angle change during the process of knee joint flexion or extension, that is, the absolute difference between two adjacent extrema. The average angular velocity refers to the ratio of the range of joint angle change to the time taken during knee joint flexion or extension.

### 2.4 Statistical analyses

Descriptive statistics are reported as the Mean ± Standard deviation. The Shapiro–Wilk test was used to determine normality. Non-normal data were logarithmically transformed prior to subsequent analysis (17), including the angular velocity of the second knee flexion during the approach phase, the angular velocity of the second knee flexion and extension during the support phase, and the ROM of knee flexion during the swing phase. A partial correlation analysis, with sex as a control variable, was conducted to examine the relationship between knee joint kinematics and kicking distances. The correlation coefficients are interpreted as follows: 0.1 ≤ |*r*| < 0.3 indicates a low correlation, 0.3 ≤ |*r*| < 0.5 indicates a moderate correlation, and 0.5 ≤ |*r*| ≤ 1 indicates a high correlation (18). The 95% confidence intervals (CI) for the correlation coefficients were calculated using Fisher's *z* transformation (19). The significance level was set at *p* < 0.05.

## 3 Results

**Figure 4:**
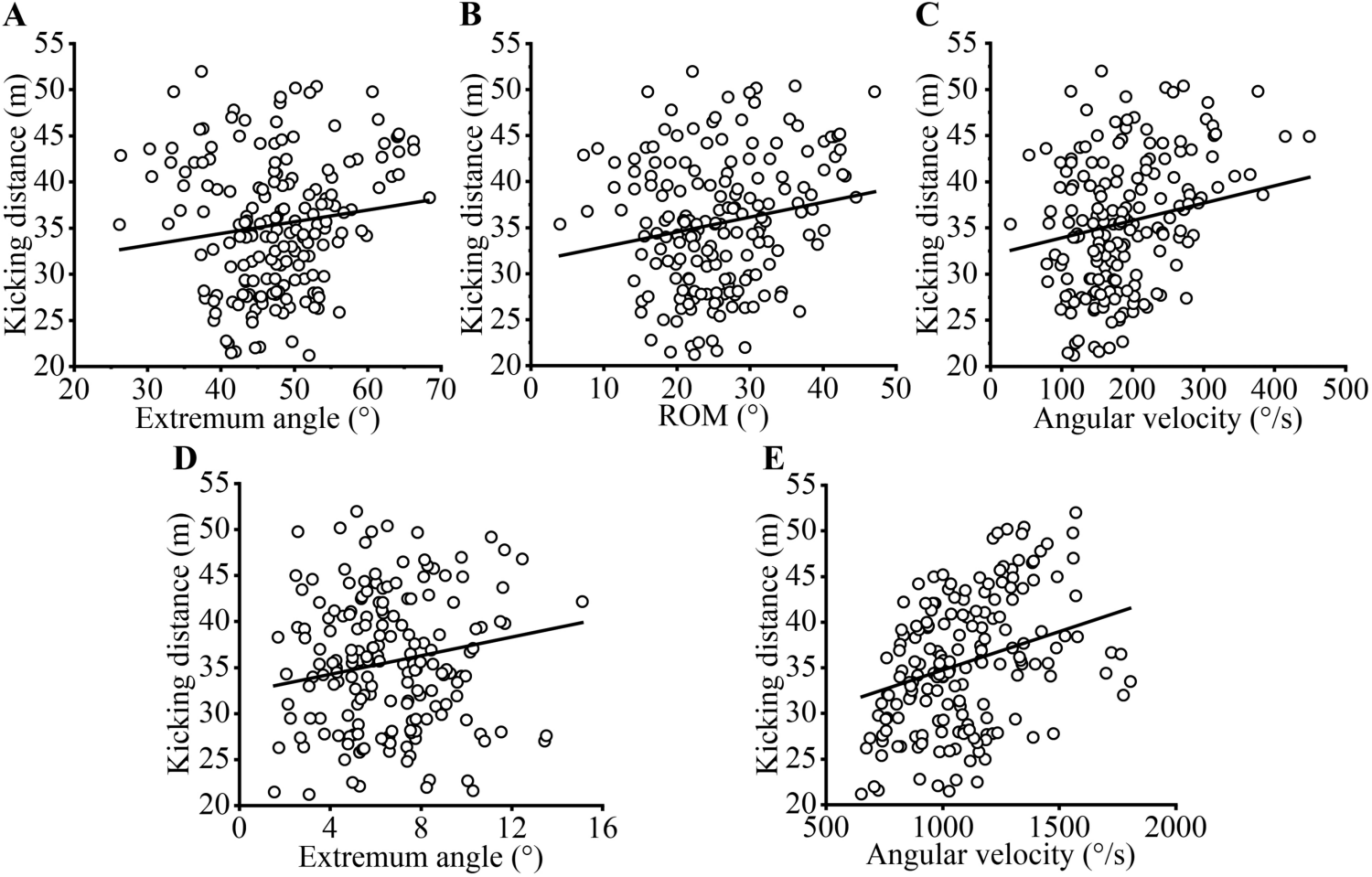
Scatter plots for significant correlations; **(A)** the correlation between the extremum angle of the second knee flexion during the approach phase and the kicking distance; **(B,C)** the correlations between the ROM and the average angular velocity of the second knee extension during the approach phase, and the kicking distance; **(D,E)** the correlation between the extremum angle and the average angular velocity of the second knee extension during the swing phase, and the kicking distance.

The results indicate that the average kicking distance for soccer players was 35.49 ± 7.18 m. Table 1 presents the kinematic parameters of the knee joint during the kicking process, including the extremum angle, ROM, and average angular velocity at different time points during the approach, support, and swing phases. Table 2 and Figure 4 present the results of the correlation analysis between the kinematic parameters of the knee joint and the kicking distance. During the approach phase, the extremum angle of the second knee flexion (*r* = 0.152, *p* = 0.041; Figure 4A), as well as the ROM of the second knee extension (*r* = 0.169, *p* = 0.023; Figure 4B) and the average angular velocity of the second knee extension (*r* = 0.185, *p* = 0.013; Figure 4C), were positively correlated with the kicking distance. During the swing phase, the extremum angle (*r* = 0.178, *p* = 0.016; Figure 4D) and the average angular velocity (*r* = 0.283, *p* < 0.001; Figure 4E) of knee extension were positively correlated with the kicking distance.

**Table 1:** Kinematic parameters of the knee joint during kicking (*n* = 26).

Phases	Knee joint	Extremum angle (°)	ROM (°)	Angular Velocity (°/s)
Approach	1st KF	70.64 ± 9.31	44.23 ± 10.74	224.48 ± 54.88
	1st KE	40.85 ± 8.17	29.79 ± 6.67	204.82 ± 56.10
	2nd KF	47.59 ± 7.84	6.74 ± 2.96	116.76 ± 83.85
	2nd KE	21.83 ± 4.21	25.76 ± 8.06	186.90 ± 70.22
Support	1st KF	68.00 ± 11.17	39.85 ± 14.84	261.35 ± 91.79
	1st KE	25.30 ± 5.71	42.70 ± 10.55	263.32 ± 65.20
	2nd KF	34.99 ± 6.39	9.69 ± 4.62	123.25 ± 68.81
	2nd KE	23.17 ± 5.35	11.85 ± 5.64	154.94 ± 99.55
Swing	KF	102.71 ± 6.16	77.04 ± 12.63	437.11 ± 108.87
	KE	6.58 ± 2.55	96.13 ± 6.29	1,092.23 ± 241.37

KF, knee flexion; KE, knee extension; ROM, range of motion.

**Table 2:** Partial correlation analysis results between knee joint kinematic parameters and kicking distance (controlled variable: sex).

Phases	Knee joint	Extremum angle			ROM			Angular velocity		
		*r*	95% CI	*p*	*r*	95% CI	*p*	*r*	95% CI	*p*
Approach	1st KF	0.122	(−0.024, 0.263)	0.102	−0.104	(−0.246, 0.043)	0.162	−0.084	(−0.227, 0.063)	0.260
	1st KE	0.077	(−0.070, 0.220)	0.303	0.030	(−0.116, 0.175)	0.692	0.011	(−0.135, 0.157)	0.884
	2nd KF	**0** **.** **152**	**(0.006 0.291)**	0.041*	0.094	(−0.053, 0.237)	0.206	0.116	(−0.030, 0.258)	0.118
	2nd KE	−0.038	(−0.183, 0.108)	0.609	**0** **.** **169**	**(0.024, 0.307)**	0.023*	**0** **.** **185**	**(0.040, 0.322)**	0.013*
Support	1st KF	0.127	(−0.019, 0.268)	0.089	0.031	(−0.115, 0.176)	0.681	0.062	(−0.085, 0.206)	0.411
	1st KE	0.095	(−0.052, 0.238)	0.202	0.082	(−0.065, 0.225)	0.270	0.062	(−0.085, 0.206)	0.408
	2nd KF	0.017	(−0.129, 0.162)	0.822	−0.127	(−0.268, 0.019)	0.088	−0.111	(−0.253, 0.035)	0.137
	2nd KE	−0.008	(−0.154, 0.138)	0.915	0.074	(−0.073, 0.218)	0.322	0.129	(−0.017, 0.270)	0.083
Swing	KF	0.134	(−0.012, 0.274)	0.072	0.037	(−0.109, 0.182)	0.621	0.053	(−0.094, 0.197)	0.478
	KE	**0** **.** **178**	**(0.033, 0.316)**	0.016*	0.071	(−0.076, 0.215)	0.341	**0** **.** **283**	**(0.143, 0.412)**	<0.001**

The significance is highlighted in bold.

∗ *p* < 0.05. ∗∗*p* < 0.01.

## 4 Discussion

The present study examined the relationship between players’ knee joint kinematics and kicking distance. It was found that during the approach phase, the maximum angle of the second knee flexion, as well as the ROM and average angular velocity of the second knee extension, were positively correlated with the kicking distance. The approach phase is the technical action process that is completed in the form of running. Players use this phase to generate more momentum, which is then transferred to the football, aiding in better control of the ball's velocity and direction, and increasing the power of passes or shots (6). During this phase, the second knee flexion occurs during the touchdown process at the end of the approach. The knee joint buffers the ground reaction force through flexion and utilizes the stretch-shortening cycle mechanism. The quadriceps undergo eccentric contraction to store elastic potential energy for the subsequent extension (20). This also promotes an increase in the ROM and average angular velocity of the second knee extension. The rapid and full extension of the knee effectively enhances the horizontal propulsion force away from the ground. This force is ultimately converted into the overall kinetic energy of the kicking motion, thereby helping players kick the football to a greater flying distance (21). Previous studies have shown that approaching the football with a faster running velocity increases the ball's exit velocity (22, 23), which is consistent with the findings of this study.

The support phase is equally critical to the performance of kicking technique. Upon landing, the supporting leg cushions the horizontal velocity acquired from the approach, converting it into vertical velocity for the body's upward motion, while simultaneously withstanding ground reaction forces equivalent to 2–3 times the player's body weight (24, 25). However, the results of this study reveal that there is no correlation between the kinematic characteristics of the knee joint during the support phase and the kicking distance. We speculate that this could be due to two factors: firstly, the high level of athleticism exhibited by all players, who executed this phase of the movement adequately; secondly, the study's protocol required players to perform the kicking technique with only a single step approach, being a short distance and may not fully reveal the impact of the support phase on kicking distance. Previous research has indicated that extending the approach distance can lead to a further acceleration in the ball velocity during a kick (26). Nevertheless, it remains uncertain whether this enhancement is linked to the kinematics of the knee joint. Therefore, future research should incorporate a larger sample size to further investigate the relationship between the kinematic characteristics of the knee joint during the support phase and kicking distance across varying approach distances.

The swing phase refers to the swinging motion of the kicking leg as it completes the striking motion, primarily achieved through the flexion and extension of lower limb joints. This study found that soccer players with a faster extension velocity of the swinging knee tend to achieve a longer kicking distance. This finding is consistent with previous research, indicating that knee extension ability is an important factor in kicking performance (1). Related electromyographic studies suggest that, although the specific muscle tissues have not been clearly identified, the knee extensor muscles play a likely crucial role in enhancing foot velocity during the striking motion (27). However, other research has indicated that the hamstrings undergo eccentric contraction during the forward swing, reach their maximum length, thereby increasing the risk of injury (28, 29). Additionally, the results of this study show that players who achieve a longer kicking distance have a greater degree of knee flexion at the final stage of extension (in this study, a fully extended knee joint is defined as 0°). This may be due to the posterior thigh muscles being passively stretched and then contracting before the ball strike as the lower leg accelerates forward, to control the knee joint angle, thereby improving the accuracy of ball contact.

This study provides valuable insights for optimizing training methods by clearly identifying key knee joint kinematic features associated with longer kicking distances. Coaches and strength and conditioning specialists can utilize these findings to design more targeted training programs. Specifically, it is essential to enhance lower limb stability, enabling greater knee flexion angles during ground contact in the approach phase while maintaining safe and efficient absorption of ground reaction forces. Furthermore, the role of knee extension during both the push-off phase of the approach and the swing-kick motion highlights the importance of incorporating knee extension-focused exercises into explosive strength training. Additionally, complementary flexibility and coordination exercises should be integrated to maximize leg movement velocity and optimal kicking angles.

This study still has some limitations that need to be addressed. This study was conducted on an outdoor football field, which ensured some ecological validity. However, we limited the analysis to single-step approaches, although this limitation also existed in previous studies (30). Nevertheless, in most cases, players tend to use multi-step approaches, which means the ecological validity of this study is somewhat lacking at this level. Additionally, this study did not assess the movement variability of the players, focusing only on the average kinematic characteristics of the movements. While this helps identify common patterns in technical execution, it may overlook individual differences in their ability to adapt through variability in dynamic environments (31). Future research could quantify the variability in players’ kicking techniques to gain a more comprehensive understanding of the adaptive mechanisms underlying their movement control. Furthermore, although previous studies have validated the effectiveness of the IMU combined with OpenSim method against a gold standard (13), there is a lack of data specifically from kicking movements. Future research could conduct confirmatory studies in this regard and include more football-specific technical scenarios to support the application of this method in football science research. Future studies could also further reveal the biomechanical mechanisms influencing kicking distance and accuracy by including a wider range of sample sizes, measuring additional joints, or focusing on parameters related to ball contact quality.

## 5 Conclusion

This study, which utilized IMUs and OpenSim modeling, analyzed the correlation between knee joint kinematics in soccer players and their kicking distances. The study found that a larger flexion angle during the ground contact phase of the approach, a faster extension velocity and a greater range of motion during the push-off phase, as well as a faster extension velocity and a greater final flexion angle during the swing phase, were associated with longer kick distances.

## Data Availability

The raw data supporting the conclusions of this article will be made available by the authors, without undue reservation.
